# Relamorelin in Gastroparesis and Diabetic Gastroparesis: A Meta-Analysis on Its Efficacy and Safety

**DOI:** 10.7759/cureus.48303

**Published:** 2023-11-05

**Authors:** Akash Patel, Gagandeep Singh Arora, Mona Roknsharifi, Hamna Javed, Parneet Kaur

**Affiliations:** 1 Internal Medicine, Eisenhower Health, Rancho Mirage, USA; 2 Hepatobiliary Pancreatic Surgery and Liver Transplant, BLK-Max Super Speciality Hospital, New Delhi, IND; 3 Internal Medicine, University of California, Riverside, San Bernardino, USA; 4 Internal Medicine, Saint Agnes Medical Center, Fresno, USA; 5 Emergency, Civil Hospital Mukerian, Mukerian, IND; 6 Internal Medicine, Suburban Community Hospital, Philadelphia, USA

**Keywords:** systematic review and meta-analysis, randomized controlled trial, gastroparesis treatment, relamorelin, gastric emptying, diabetic gastroparesis (dg), gherlin receptor agonist, impaired gastric motility

## Abstract

This meta-analysis aimed to evaluate the efficacy and safety of relamorelin, a synthetic ghrelin receptor agonist, for the treatment of gastroparesis and diabetic gastroparesis. A total of 1,033 participants from five randomized controlled trials were included. The primary outcome was the mean change in gastric emptying time from baseline. Relamorelin demonstrated a statistically significant improvement in gastric emptying time with a mean difference of -11.40 minutes compared to the placebo group. Furthermore, a significant improvement was observed specifically in diabetic gastroparesis patients, with a mean difference of -8.43 minutes. However, adverse effects, such as headaches, dizziness, and gastrointestinal symptoms, were more prevalent in the relamorelin group. Despite these adverse effects, the study concludes that relamorelin offers a promising avenue for gastroparesis treatment, especially given the limited existing therapeutic options. This comprehensive meta-analysis synthesizes existing evidence to inform clinical practice and guides future research in this domain.

## Introduction and background

Gastroparesis, a debilitating gastrointestinal disorder, is characterized by delayed gastric emptying in the absence of mechanical obstruction. The condition represents a substantial medical burden, causing a wide array of symptoms such as nausea, vomiting, bloating, and early satiety, thereby significantly compromising patients' quality of life [1]. This disorder particularly places a heavy toll on healthcare systems owing to dietary modifications and an array of diagnostic tests. Despite its impact, therapeutic options remain limited and often unsatisfactory. Currently, metoclopramide is the only FDA-approved medication specifically for gastroparesis. However, its use is restricted due to a black box warning, recommending it for less than three months of treatment [1]. This paucity of treatment options highlights a significant unmet medical need for effective therapeutic strategies [2,3].

The search for novel agents has turned the spotlight onto ghrelin, a hormone predominantly found in the stomach, which serves as a natural ligand for the ghrelin or growth hormone secretagogue (GHS 1a receptor) [4]. This receptor is implicated in a wide range of physiological processes, including gastric motility. Synthetic ghrelin agonists are being developed as promising prokinetic agents and may offer utility in managing disorders of gastrointestinal motility [5]. Relamorelin, a synthetic pentapeptide amide, has emerged as a ghrelin-receptor agonist with enhanced potency, plasma stability, and circulating half-life [6]. In light of its favorable pharmacokinetics and pharmacodynamics, relamorelin is increasingly being considered a potential candidate for gastroparesis treatment.

Although relamorelin has been studied in several randomized controlled trials (RCTs), no comprehensive meta-analysis has hitherto been conducted to evaluate its efficacy and safety. This study aims to fill this gap by conducting a meta-analysis of available RCTs that have investigated the role of relamorelin in improving gastric emptying, with a special focus on diabetic gastroparesis patients. The safety profile of relamorelin, including adverse effects such as hyperglycemia and gastrointestinal symptoms, is also critically assessed. 

Thus, this meta-analysis seeks to provide a holistic understanding of the efficacy and safety of relamorelin and synthesizes the existing evidence to inform clinical practice and future research directions.

## Review

Methods

Data Sources and Search Strategy

To assemble a thorough compilation of relevant studies, systematic searches were conducted across multiple electronic databases, including MEDLINE, PubMed, Embase, Web of Science, and Google Scholar. Reference lists of pertinent articles were additionally examined to augment the search process.

The search was centered on the term "Relamorelin," coupled with associated keywords such as "gastric motility," "gastric emptying," "gastroparesis," "diabetic gastroparesis," "adverse effects," "side effects," "complications," and "safety." The last search was performed on October 1, 2023.

Eligibility Criteria

The inclusion criteria necessitated studies to be placebo-controlled, randomized controlled trials (RCTs) featuring relamorelin as the primary intervention. No restrictions were placed on dosage levels. Studies needed to primarily or secondarily investigate relamorelin’s effect on gastric emptying time and adverse effects and be peer-reviewed. Exclusion criteria consisted of non-RCT designs, unpublished works, non-English language publications, and studies deviating from the central research focus. Reviews, editorials, case reports, and commentaries were also excluded.

Study Selection and Data Extraction

Initially, the titles and abstracts of the 560 identified articles were independently reviewed by two investigators. For this screening process, a combination of tools was employed, including 'Publish or Perish' for literature search and retrieval, EndNote for reference management, and Microsoft Excel for organizing and filtering the initial findings. Additionally, manual searches were conducted to ensure comprehensive coverage and to cross-verify the electronic search results. Subsequently, full-text evaluations were conducted to confirm each study's relevance and methodological congruence. Data were then systematically extracted by two independent researchers, encapsulating variables such as demographic details, baseline characteristics, geographical settings, dosages, and durations, along with primary and secondary outcomes.

Risk of Bias Assessment

The risk of bias was evaluated using the Cochrane risk of bias tool (RoB 2.0), tailored for RCTs, by two investigators. Multiple domains were assessed, including selection, performance, detection, attrition, and reporting biases. Each study was categorized as 'low risk,' 'high risk,' or 'unclear risk' in each domain. Disagreements were resolved through consensus following structured discussions between two independent investigators.

Statistical Analysis

The primary endpoint for this meta-analysis was the mean change in gastric emptying time from baseline, utilizing a linear distribution assumption and considering the least squares (LS) mean as the mean value. Data were continuous in nature, and the effect measure employed was the mean difference (MD) between the relamorelin and placebo groups. Adverse effects were examined in a descriptive manner, focusing solely on the frequency of events across studies. Heterogeneity among included studies was assessed using the I^2^ statistic, with values categorized as low, moderate, or high for I^2^ values of 25%, 50%, and 75%, respectively. In case of significant heterogeneity, a random-effects model was applied for the pooled analysis. The inverse variance method was used for pooling the effect sizes. Results were reported with a 95% confidence interval (CI). Statistical significance was set at p < 0.05, and all calculations were executed using RevmanWeb.

Results

Study Selection and Characteristics

**Figure 1:**
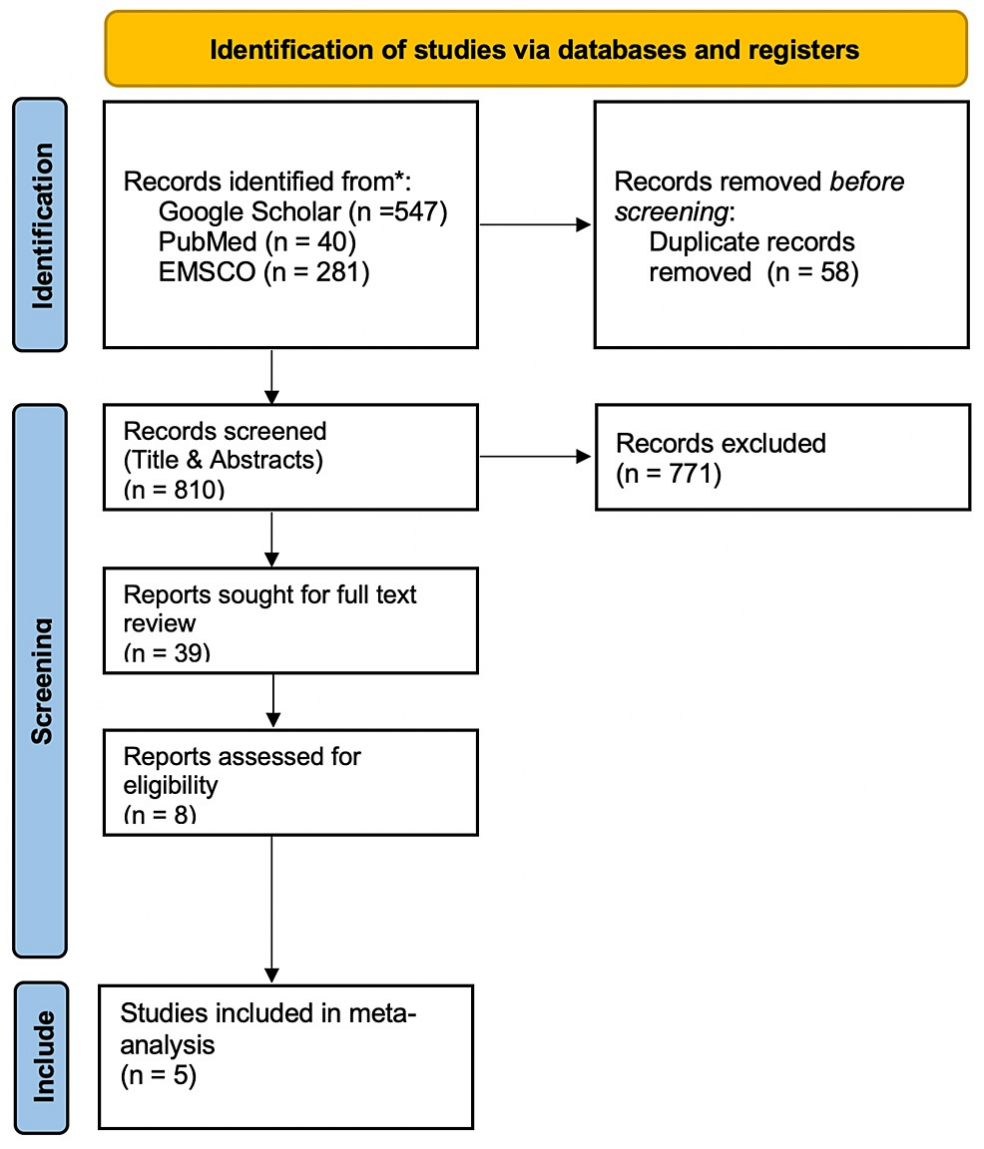
PRISMA Flowchart

The initial database identified a total of 868 publications. After duplicates were removed, 810 articles underwent abstract review, as shown in Figure 1. Subsequently, 39 articles underwent full-text review, out of which five met the inclusion criteria for this meta-analysis. The five included RCTs were by Acosta et al. [7], Lembo et al. [8], Camilleri et al. [9], Camilleri et al. [10], and Fazeli et al. [11]. Detailed study selection and characteristics are summarized in Figure 1 and Table 1, respectively.

**Table 1:** Study Characteristics QD: Once Daily, BID: Twice Daily, GEBT: Gastric Emptying Breath Test, AEs: Adverse Events, t1/2: Gastric Emptying Half-time, GCSI-DD: Gastroparesis Cardinal Symptom Index - Daily Diary, PAGI-SYM: Patient Assessment of Gastrointestinal Symptoms, s.c.: Subcutaneous Injection, b.i.d.: Twice Daily (alternative form of BID), N/A: Not Applicable

Study Characteristic	Acosta et al. [7]	Lembo et al. [8]	Camilleri et al. [9]	Camilleri et al. [10]	Fazeli et al. [11]
Design Type	Randomized, double-blind, placebo-controlled, parallel-group	28-day, randomized, double-blind, placebo-controlled	Randomized, double-blind, placebo-controlled phases 2a and 2b	12-week, randomized, double-blind, placebo-controlled, parallel-group	Randomized, double-blind, placebo-controlled
Centers	2-center (Mayo Clinic)	27 clinical centers in the U.S.	Multiple clinical centers	U.S., Israel, and Europe	Massachusetts General Hospital
Age Range	18-65 years	18-75 years	18-75 years	18-75 years	Not specified
Patient Characteristics	Chronic constipation	Diabetic gastroparesis	Diabetic gastroparesis	Diabetic gastroparesis	Anorexia nervosa with GI symptoms
Drug & Dosage	Relamorelin (100 μg QD)	Relamorelin (10 μg BID, 10 μg QD)	Relamorelin (10 µg, 30 µg, 100 µg b.i.d.) s.c.	Relamorelin (10 μg, 30 μg, 100 μg) b.i.d. s.c.	Relamorelin (100 μg QD)
Primary Endpoints	Colonic transit at 24h, stool consistency	Gastric emptying half-time (t1/2)	Adverse events, treatment-emergent AEs	Vomiting frequency	Weight change over 4 weeks
Secondary Endpoints	Gastric emptying half-time (t1/2)	Daily symptoms of diabetic gastroparesis	Injection site assessments, weight changes, vital signs	Vomiting severity, GEBT T1/2, individual symptoms, GCSI-DD score	Resting energy expenditure, GEBT results, depression, and PAGI-SYM scores
Method for Gastric Emptying Study	Scintigraphic method	13C-spirulina GEBT	N/A	13C-spirulina GEBT	GEBT with 13C-spirulina platensis meal
Timeframe for Gastric Emptying	End of 14-day treatment	On day 28	N/A	Baseline and at 12 weeks	Baseline and week 4

Participant Characteristics

In total, the five studies comprised 1,033 participants, with 482 in the placebo group and 551 in the relamorelin group. The ages of the participants ranged from 18 to 75 years. The percentage of female participants varied between the studies but was generally high. A detailed breakdown of participant characteristics is shown in Table 2.

**Table 2:** Participant Characteristics Across Studies

Characteristic	Study Name	Placebo	Relamorelin
Total Participants (N)	Acosta et al. [7]	23	25
	Lembo et al. [8]	69	135
	Camilleri et al. [9]	104	289
	Camilleri et al. [10]	104	295
	Fazeli et al. [11]	12	10
Mean Age (years)	Acosta et al. [7]	41	40
	Lembo et al. [8]	55	55
	Camilleri et al. [9]	Overall study 57	Overall study 57
	Camilleri et al. [10]	55.7	57.5
	Fazeli et al. [11]	29	29
% Female Participants	Acosta et al. [7]	100%	100%
	Lembo et al. [8]	68.1%	66.7%
	Camilleri et al. [9]	Overall study 62%	Overall study 62%
	Camilleri et al. [10]	64%	60%
	Fazeli et al. [11]	100%	100%

Quality and Risk of Bias

All studies were of moderate to high quality, with well-defined random sequence generation and allocation concealment in most cases. The risk of bias for each study is summarized in Table 3.

**Table 3:** Risk of Bias Across Studies

Bias Domain	Acosta et al. [7]	Lembo et al. [8]	Camilleri et al. [9]	Camilleri et al. [10]	Fazeli et al. [11]
Random sequence generation (selection bias)	Low: Well-defined	Low: Well-defined	Low: Well-defined	Low: Well-defined	Unclear: Not specified
Allocation concealment (selection bias)	Low: Well-concealed	Low: Well-concealed	Unclear: Not specified	Low: Well-concealed	Unclear: Not specified
Blinding of participants and personnel (performance bias)	Low: Double-blind	Low: Double-blind	Low: Double-blind	Low: Double-blind	Low: Double-blind
Blinding of outcome assessment (detection bias)	Low: Outcome blinded	Unclear: Not specified	Low: Outcome blinded	Low: Outcome blinded	Low: Likely blinded
Incomplete outcome data (attrition bias)	Unclear: No details	Unclear: No details	Unclear: No details	Unclear: No details	Low: ITT analysis
Selective reporting (reporting bias)	Low: Registered	Low: All endpoints	Low: Registered	Unclear: Incomplete	Low: Registered

Effectiveness of Relamorelin on Gastric Emptying Improvement From Baseline

Our analysis was focused on the effectiveness of relamorelin on gastric emptying, and evidence predominantly suggests a favorable impact. Camilleri et al. [10] reported a 13-minute improvement in gastric emptying half-time (t1/2) among patients treated with 100 µg QD of relamorelin. Similarly, Lembo et al. [8] demonstrated an almost 15-minute reduction in t1/2 in diabetic gastroparesis patients administered 10 µg BID or QD of the drug. Additionally, Fazeli et al. [11] demonstrated a 31-minute improvement in gastric emptying time from baseline.

**Figure 2:**
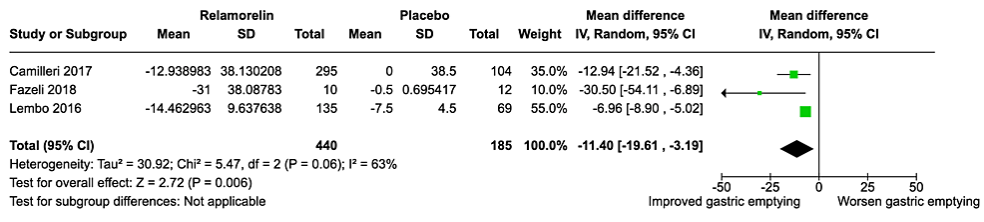
Effectiveness of Relamorelin on Gastric Emptying Improvement From Baseline With Mean Difference

The overall MD for change in gastric emptying from baseline favored relamorelin over placebo, with an MD of -11.40 (95% CI: -19.61, -3.19) (Figure 2). The heterogeneity of the pooled studies was moderate, with I² = 63%. The Z-test for the overall effect was significant (Z = 2.72, P = 0.006).

Diabetic Gastroparesis on Gastric Emptying Improvement From Baseline

**Figure 3:**
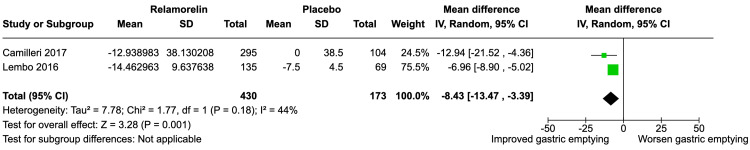
Effectiveness of Relamorelin on Gastric Emptying Improvement From Baseline (Study Focused on Diabetic Gastroparesis)

For diabetic gastroparesis patients, relamorelin showed a statistically significant improvement in gastric emptying over placebo, with a mean difference of -8.43 (95% CI: -13.47, -3.39) (Figure 3). The heterogeneity among these studies was low to moderate (I² = 44%), and the Z-test for overall effect was significant (Z = 3.28, P = 0.001).

Safety and Adverse Effects

The safety profile of relamorelin was thoroughly evaluated across the studies, and Table 4 summarizes the adverse effects reported.

**Table 4:** Adverse Effects Across Studies

Study	Group	Adverse Effect Subgroups	Counts
Acosta et al. [7]	Placebo	General Symptoms	
		Headaches & Dizziness	7 (Headache) + 3 (Lightheaded)
		Gastrointestinal Symptoms	
		Common GI Symptoms	6 (Bloating) + 4 (Nausea) + 4 (Abdominal ache)
	Relamorelin	General Symptoms	
		Headaches & Dizziness	16 (Headache) + 5 (Lightheaded)
		Fatigue & Sinus Issues	7 (Fatigue) + 5 (Sinus congestion)
		Gastrointestinal Symptoms	
		Common GI Symptoms	10 (Bloating) + 5 (Nausea) + 7 (Abdominal ache)
Lembo et al. [8]	Placebo	General Symptoms	
		Headaches & Dizziness	2 (Headache) + 4 (Dizziness)
	Relamorelin Combined	General Symptoms	
		Headaches & Dizziness	2 (Relamorelin once daily) + 5 (Relamorelin twice daily) (Headache) + 1 (Relamorelin once daily) + 1 (Relamorelin twice daily) (Dizziness)
		Infections	
		UTI	2 (Relamorelin once daily) + 2 (Relamorelin twice daily)
Camilleri et al. [10], Camilleri et al. [9]	Placebo	General Symptoms	
		Headaches & Dizziness	3 (Headache) + 1 (Dizziness)
	Relamorelin Combined	General Symptoms	
		Headaches & Dizziness	4 (Relamorelin 10 mcg) + 6 (Relamorelin 30 mcg) + 2 (Relamorelin 100 mcg) (Headache) + 0 (Relamorelin 10 mcg) + 1 (Relamorelin 30 mcg) + 5 (Relamorelin 100 mcg) (Dizziness)
		Gastrointestinal Symptoms	
		GI Discomfort	0 (Relamorelin 10 mcg) + 3 (Relamorelin 30 mcg) (Constipation) + 6 (Relamorelin 10 mcg) + 18 (Relamorelin 30 mcg) + 13 (Relamorelin 100 mcg) (GI symptoms) + 4 (Relamorelin 10 mcg) + 7 (Relamorelin 30 mcg) + 6 (Relamorelin 100 mcg) (Diarrhea)
		Metabolic & Infections	
		Diabetes & Infections	2 (Relamorelin 10 mcg) + 1 (Relamorelin 30 mcg) + 3 (Relamorelin 100 mcg) (Diabetes) + 7 each from Relamorelin 10 mcg, 30 mcg, and 100 mcg (UTI) + 1 each from Relamorelin 10 mcg, 30 mcg, and 100 mcg (DKA)

General symptoms: Headaches and dizziness were more prevalent in the relamorelin group across the studies. Specifically, the study by Acosta et al. [7] reported 16 cases of headaches and five cases of lightheadedness in the relamorelin group compared to seven and three, respectively, in the placebo group. A similar trend was observed by Lembo et al. [8], where both relamorelin once daily and twice daily groups reported more cases of headaches and dizziness compared to the placebo group.

Gastrointestinal symptoms: Common gastrointestinal symptoms, such as bloating, nausea, and abdominal aches, were noted in all studies but appeared slightly more frequent in the relamorelin group. For instance, in Acosta et al. [7], 10 cases of bloating, five of nausea, and seven of abdominal aches were reported in the relamorelin group as compared to six, four, and four in the placebo groups, respectively. 

Infections: Urinary tract infections (UTIs) were documented by Lembo et al. [8] and the combined data of Camilleri et al. [9,10]. Specifically, in Lembo et al. [8], two cases each of UTI were reported for both relamorelin once and twice daily groups. Similarly, seven cases each from all dosage groups were reported for UTI in the combined data of Camilleri et al. [9,10].

Metabolic concerns: Diabetic ketoacidosis (DKA) and diabetes were reported only in the combined data of Camilleri et al. [9,10] but were relatively rare. The data showed one case each of DKA from all relamorelin dosage groups.

Discussion

Gastroparesis is a gastrointestinal disorder characterized by delayed gastric emptying in the absence of mechanical obstruction, and it poses a significant burden on patients and healthcare systems alike [1]. The current pharmacotherapeutic options are limited, with metoclopramide being the only FDA-approved medication for gastroparesis. However, its usage is hampered by a black box warning recommending its use for less than three months [1]. This represents a significant unmet medical need, thereby making the search for effective treatments crucial [2,3].

Our meta-analysis included a total of five studies with 1,033 participants, focusing on the efficacy and safety of Relamorelin in improving gastric emptying. Three studies - Camilleri et al. [10], Fazeli et al. [11], and Lembo et al. [8]- were pivotal for our main analysis, which aimed to evaluate the mean difference for change in gastric emptying from baseline when compared to a placebo group. The pooled results indicated a statistically significant mean difference of -11.40 minutes (95% CI: -19.61, -3.19), suggesting that relamorelin may be effective in accelerating gastric emptying.

In terms of molecular targets, relamorelin acts as a ghrelin receptor agonist. Ghrelin is a hormone found predominantly in the stomach and is involved in regulating several aspects of gastrointestinal motility [4]. In particular, relamorelin was found to be ~sixfold more potent than human ghrelin in activating ghrelin receptors and ~100-fold more potent in terms of its overall effect [12]. This suggests that relamorelin's pronounced efficacy could be attributed to its potent activation of ghrelin receptors, both in the gut and the CNS [13].

Subgroup analyses were conducted to evaluate the efficacy of relamorelin specifically in diabetic gastroparesis, including studies by Lembo et al. [8] and Camilleri et al. [10]. The findings were consistent with the main analysis, showing a statistically significant mean difference of -8.43 minutes (95% CI: -13.47, -3.39). This is noteworthy given the paucity of effective treatment options for diabetic gastroparesis [10].

Regarding safety, our analysis included four studies that reported adverse effects. Notably, one of the key adverse effects associated with relamorelin was an increased frequency of hyperglycemic episodes. This may be due to accelerated gastric emptying, resulting in quicker nutrient absorption and consequent hyperglycemia. Additionally, mild diarrhea was reported, which aligns with the known effects of ghrelin receptors in the enteric neural control of the colon [14].

While our study suggests that relamorelin is a promising prokinetic agent for treating gastroparesis, there are limitations. One such limitation is the heterogeneity across the included studies, as indicated by an I² of 63% in the main analysis. Furthermore, longer-term studies are needed to evaluate the sustainability of the therapeutic effects and to better understand the safety profile of relamorelin, particularly regarding its endocrine effects such as its stimulation of pituitary growth hormone [10].

## Conclusions

Our meta-analysis suggests that relamorelin is a promising treatment option for improving gastric emptying, particularly in patients with diabetic gastroparesis. However, caution should be exercised due to the potential for hyperglycemia and gastrointestinal side effects. Further studies, ideally randomized controlled trials with larger sample sizes and longer durations, are warranted to substantiate these findings.
